# Impaired mitophagy in Sanfilippo a mice causes hypertriglyceridemia and brown adipose tissue activation

**DOI:** 10.1016/j.jbc.2022.102159

**Published:** 2022-06-22

**Authors:** Miguel Tillo, William C. Lamanna, Chrissa A. Dwyer, Daniel R. Sandoval, Ariane R. Pessentheiner, Norah Al-Azzam, Stéphane Sarrazin, Jon C. Gonzales, Shih-Hsin Kan, Alexander Y. Andreyev, Nicholas Schultheis, Bryan E. Thacker, Charles A. Glass, Patricia I. Dickson, Raymond Y. Wang, Scott B. Selleck, Jeffrey D. Esko, Philip L.S.M. Gordts

**Affiliations:** 1 Division of Endocrinology and Metabolism, Department of Medicine, University of California, San Diego, La Jolla, California, USA; 2 Department of Cellular and Molecular Medicine, University of California, San Diego, La Jolla, California, USA; 3 Biomedical Sciences Graduate Program, University of California, San Diego, La Jolla, California, USA; 4 The Lundquist Institute for Biomedical Innovation at Harbor-UCLA Medical Center, Torrance, California, USA; 5 CHOC Children’s Hospital Orange County, Orange, California, USA; 6 Department of Pharmacology, University of California, San Diego, La Jolla, California, USA; 7 Department of Biochemistry & Molecular Biology, The Pennsylvania State University, University Park, Pennsylvania, USA; 8 TEGA Therapeutics Inc, San Diego, California, USA; 9 Department of Pediatrics, Washington University in St Louis, St Louis, Missouri, USA; 10 Division of Metabolic Disorders, Orange, California, USA; 11 Department of Pediatrics, University of California-Irvine School of Medicine, Irvine, California, USA; 12 Glycobiology Research and Training Center, University of California, San Diego, La Jolla, California, USA

**Keywords:** mucopolysaccharidoses, sulfamidase, mitochondria, dyslipidemia, hyperthermia, autophagy, BAT, brown adipose tissue, ERT, enzyme replacement therapy, eWAT, epididymal white adipose tissue, FFA, free fatty acid, GAG, glycosaminoglycan, LPL, lipoprotein lipase, LSD, lysosomal storage disorders, MPS, mucopolysaccharidosis, sWAT, subcutaneous white adipose tissue, TG, triglyceride, TRL, triglyceride-rich lipoprotein, VLDL, very-low-density lipoprotein

## Abstract

Lysosomal storage diseases result in various developmental and physiological complications, including cachexia. To study the causes for the negative energy balance associated with cachexia, we assessed the impact of sulfamidase deficiency and heparan sulfate storage on energy homeostasis and metabolism in a mouse model of type IIIa mucopolysaccharidosis (MPS IIIa, Sanfilippo A syndrome). At 12-weeks of age, MPS IIIa mice exhibited fasting and postprandial hypertriglyceridemia compared with wildtype mice, with a reduction of white and brown adipose tissues. Partitioning of dietary [^3^H]triolein showed a marked increase in intestinal uptake and secretion, whereas hepatic production and clearance of triglyceride-rich lipoproteins did not differ from wildtype controls. Uptake of dietary triolein was also elevated in brown adipose tissue (BAT), and notable increases in beige adipose tissue occurred, resulting in hyperthermia, hyperphagia, hyperdipsia, and increased energy expenditure. Furthermore, fasted MPS IIIa mice remained hyperthermic when subjected to low temperature but became cachexic and profoundly hypothermic when treated with a lipolytic inhibitor. We demonstrated that the reliance on increased lipid fueling of BAT was driven by a reduced ability to generate energy from stored lipids within the depot. These alterations arose from impaired autophagosome–lysosome fusion, resulting in increased mitochondria content in beige and BAT. Finally, we show that increased mitochondria content in BAT and postprandial dyslipidemia was partially reversed upon 5-week treatment with recombinant sulfamidase. We hypothesize that increased BAT activity and persistent increases in energy demand in MPS IIIa mice contribute to the negative energy balance observed in patients with MPS IIIa.

Proteoglycans are a subset of glycoproteins with the common characteristic of containing one or more covalently attached glycosaminoglycan (GAG) chains. GAG chains are long linear polysaccharides that interact with a plethora of soluble and membrane-associated proteins important in development and physiology (1, 2, 3). Cells internalize plasma membrane proteoglycans by endocytosis, resulting in delivery to the lysosome where degradation of the core protein and the GAG chains occurs. Degradation of the GAG chains occurs sequentially from the nonreducing end of the chains. Disruption of any of the enzymes involved in GAG catabolism leads to lysosomal accumulation (also called storage) of partially degraded polysaccharide causing the mucopolysaccharidoses (MPS) (4). The Sanfilippo syndromes result from defects specifically in heparan sulfate catabolism (MPS IIIA-E) and cause numerous pathological consequences in the brain and other parts of the body (4, 5).

Lysosomal GAG accumulation has a significant impact on whole body energy homeostasis (6, 7). One theory posits that storage results in lysosomal dysfunction and impairs efficient recycling of monosaccharides normally generated by the degradation of glycans in the lysosome, which serve as substrates for building new macromolecules (7). Impaired substrate turnover is believed to shift the metabolic flux within the cell toward *de novo* anabolic reactions and consequently leads to increased demand for energy from endogenous storage depots (*e.g.*, adipose tissue) or from the diet.

Triglycerides (TGs) provide a major energy source. Dietary TGs are packaged as chylomicrons in the small intestine for transport in the circulation. *De novo* synthesis of TGs occurs during fasting in the liver, giving rise to very-low-density lipoproteins (VLDLs). Chylomicrons and VLDLs undergo lipolytic processing in peripheral tissues by lipoprotein lipase, providing fatty acids for energy generation in the heart and other tissues (8). Adipocytes provide a mechanism for storing excess triglycerides, which are then mobilized during starvation (9). Recently a specialized adipocyte called the brown adipocyte has generated interest as a possible treatment for obesity because of its ability to mobilize and utilize stored lipids to generate heat by uncoupling ATP production in mitochondria (10, 11).

Mouse models of MPS display decreased liver lipid levels, reduced adiposity, and increased food consumption (6). Contrary to previous findings, we observed fasting and postprandial hypertriglyceridemia in MPS IIIa mice compared with wildtype controls (6, 7). The hypertriglyceridemia was associated with a marked increase in dietary lipid uptake in the intestine. Lipid uptake in brown adipose tissue was also elevated, fueling brown adipose tissue (BAT) thermogenesis and hyperthermia. This observation suggested that MPS IIIa knockout mice had a reduced ability to generate heat by recycling of energy substrates such as lipids and glycogen. Altered autophagy and accumulation of mitochondria was evident in adipose tissue, explaining the enhanced BAT activity. Our data provide an explanation for the increased energy demand in mouse models of MPS and possibly the negative energy balance observed in patients with MPS and corresponding animal models (12).

## Results

### MPS mice exhibit postprandial dyslipidemia

**Figure 1:**
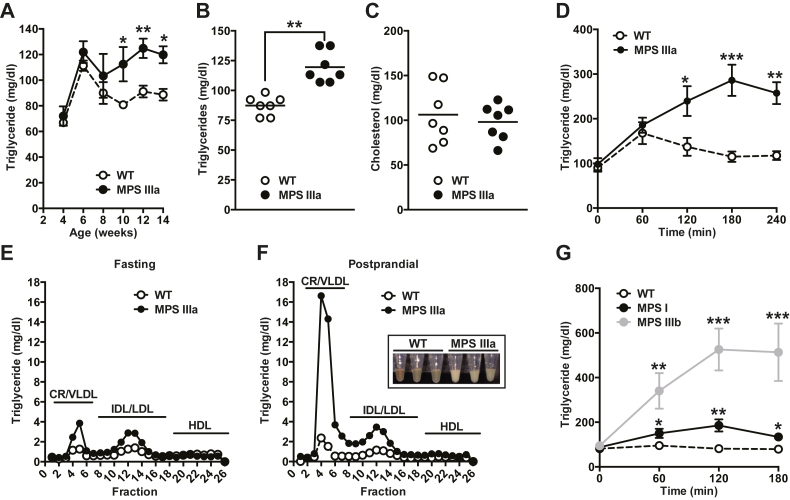
**MPS IIIa mice present with postprandial hypertriglyceridemia.***A* and *B*, fasting (5 h) plasma triglyceride (TG) levels in wildtype and MPS IIIa mice measured at different ages (n = 4 per group; *B* and *C* are derived from 12-week-old mice). *C*, fasting plasma TG and cholesterol levels were measured in 12-week-old wildtype and MPS IIIa mice. *D*, postprandial TG clearance in overnight-fasted MPS IIIa and wildtype mice after giving 200 μl of corn oil by oral gavage. TG levels were measured at the indicated time points. E-F, TG lipoprotein profiles in fasted and postprandial plasma (3 h after gavage) were analyzed by FPLC. The amount of TG in each fraction was measured. The elution positions of human chylomicron remnant (CR)/very-low-density lipoprotein (VLDL), intermediate-density lipoprotein (IDL)/low-density lipoprotein (LDL), and high-density lipoprotein (HDL) are indicated. The inset represents pictures of plasma samples 3 h after the corn oil gavage. *G*, postprandial TG clearance in overnight-fasted MPS I, MPS IIIb, and wildtype mice after giving 200 μl of corn oil by oral gavage. Each value represents the average ± SEM. ∗*p* < 0.05, ∗∗*p* < 0.01, and ∗∗∗*p* < 0.001 compared with wildtype mice.

The strain of MPS IIIa mice used in this study bears a hypomorphic allele that reduces sulfamidase activity ≥95% (13). Analysis of plasma lipids under fasting conditions showed that mutant mice exhibited hypertriglyceridemia (Fig. 1*A*), which by 12 weeks manifested as ∼40% increase in plasma TGs (120 ± 5 mg/dl in the mutant *versus*. 87 ± 5 mg/dl in the wildtype) (Fig. 1*B*). Plasma cholesterol levels did not differ (Fig. 1*C*). To test if elevated plasma TGs originated from dietary fat, fasted mice were given a bolus of corn oil by oral gavage and blood was sampled at various time points to measure the appearance and disappearance of TGs in the circulation. MPS IIIa mice exhibited impaired postprandial lipid clearance, with TG levels remaining high even 4 h post gavage (Fig. 1*D*). Analysis of plasma lipoproteins by size exclusion chromatography showed higher plasma chylomicron remnant, VLDL, and intermediate-density and low-density lipoproteins TGs both under fasted and postprandial conditions (Fig. 1, *E* and *F*). The extent of hyperlipidemia in the mutant was obvious by the cloudy nature of postprandial plasma (Fig. 1*F*, inset). A similar pre- and postprandial plasma lipid analysis in mice lacking α-*N*-acetylglucosaminidase activity (*Naglu*^-/-^; MPS IIIb) and mice lacking α-L-iduronidase activity (*Idua*^-/-^; MPS I) showed no variation in plasma TGs compared with wildtype mice (88 ± 9 mg/dl [*Naglu*^-/-^] and 95 ± 10 mg/dl [*Idua*^-/-^] *versus* 93 ± 9 mg/dl [wildtype], respectively), in agreement with previous observations (6). However, when challenged with a bolus of corn oil by oral gavage, both MPS I and MPS IIIb mice became hyperlipidemic compared with wildtype controls, with MPS IIIb showing the strongest response (Fig. 1*G*). These findings indicate that postprandial hypertriglyceridemia may be a general feature of mucopolysaccharidoses.

### MPS IIIa mice exhibit enhanced intestinal lipid absorption

**Figure 2:**
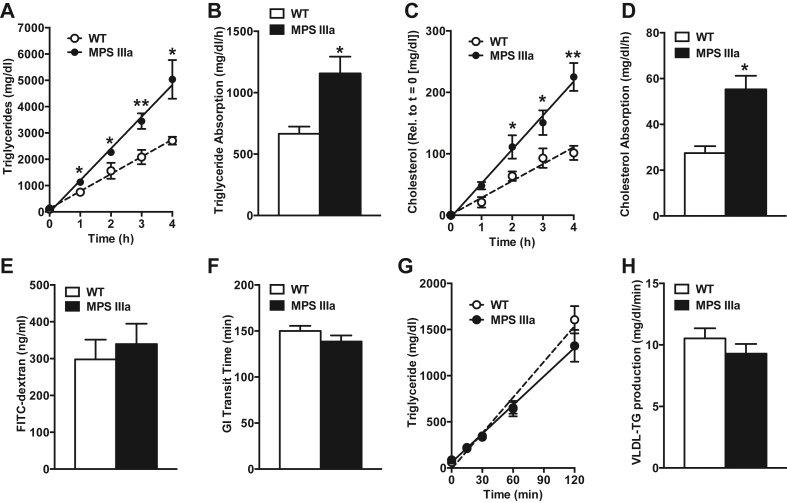
**Increased intestinal lipid absorption and secretion in MPS IIIa mice.***A*–*D*, Postprandial lipid absorption and chylomicron production was evaluated after a combined gastric corn oil load and Triton WR-1339 injection. Blood samples were collected at the indicated time points and processed to measure plasma triglyceride (A-B) and cholesterol (C-D) accumulation and calculate absorption rates (n = 4–5 per group). *E*, gastrointestinal permeability was determined in overnight-fasted wildtype and MPS IIIa mice after an oral gavage with FITC-dextran. Blood samples were drawn 4 h later and the presence of FITC-dextran was measured (n = 4–5 per group). *F*, wildtype and MPS IIIa mice received an oral gavage of carmine red. The time of red stool appearance was recorded as a measure for gastrointestinal transit time (n = 4–5 per group). G-H, Hepatic very-low-density lipoprotein production was determined in overnight-fasted wildtype and MPS IIIa mice after a Triton WR-1339 injection to inhibit lipolysis. Blood samples were collected at the indicated time points and processed to measure plasma triglyceride accumulation and calculate very-low-density lipoprotein production rates (n = 6 per group). Each value represents the average ± SEM. ∗*p* < 0.05 and ∗∗*p* < 0.01 compared with wildtype mice.

The liver produces VLDL TGs from pools of endogenous lipids, while the small intestine produces chylomicrons from dietary lipids. To assess intestinal lipid absorption and production, fasted mice were injected with Triton WR-1339 to inhibit lipolytic turnover of VLDL. The injection was subsequently followed by a bolus of corn oil *via* oral gavage, followed by periodic blood collection. Under these conditions MPS IIIa mice accumulated plasma TGs at a 1.7-fold higher rate than in wildtype mice (Fig. 2, *A* and *B*), indicative of increased intestinal lipid absorption and chylomicron production. The increased plasma TG accumulation was paralleled with similar increased rate of plasma cholesterol accumulation (2-fold; Fig. 2, *C* and *D*) indicative of increased intestinal chylomicron production. Dextran absorption did not differ in mutant and wildtype mice, excluding the possibility that lysosomal storage disease impacted intestinal integrity (Fig. 2*E*). Gastrointestinal transit time of a food bolus also did not differ (Fig. 2*F*). To test if hepatic VLDL production contributed to elevated TG levels, mice were fasted and injected with Triton WR-1339to inhibit lipolytic turnover of VLDL. Plasma VLDL TG accumulated at the same rate in MPS IIIa mice and controls (Fig. 2, *G* and *H*). These findings establish that elevated plasma TGs in MPS IIIa mice arises from enhanced dietary lipid absorption.

### Lysosomal storage does not affect lipoprotein lipase activity or VLDL clearance

**Figure 3:**
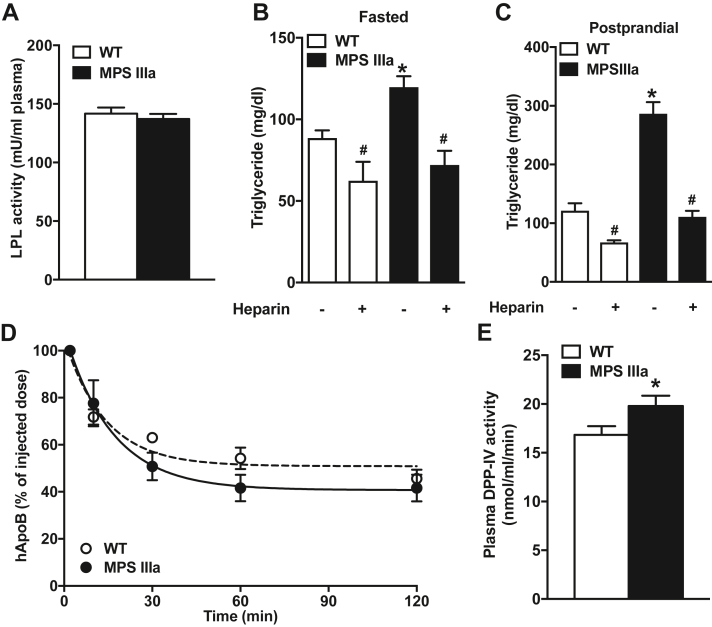
**MPS IIIa mice have normal LPL activity and VLDL clearance rates.***A*, plasma LPL activity levels were determined in overnight-fasted wildtype and MPS IIIa mice 10 min after an i.v. injection with heparin (50 U per mouse; n = 4 per group). *B* and *C*, plasma triglycerides were measured before and after heparin injection in wildtype and MPS IIIa mice after (*B*) an overnight fast or (*C*) 2 h after fat challenge. Heparin was injected intravenously (50 U per mouse) and blood was sampled *via* tail vein bleeding 10 min after injection (n = 5 per group). *D*, VLDL clearance in wildtype and MPS IIIa animals was determined after injection of 20 μg of human VLDL. At the indicated times blood samples were collected and the amount of remaining human apoB was determined by ELISA (n = 5 per group). *E*, DPP-IV activity in plasma from fasted 4-month-old wildtype and MPS IIIa mice (n = 4 per group). Each value represents the average ± SEM. ∗*p* < 0.05 compared with wildtype mice, # *p* < 0.05 compared with no heparin treated MPS IIIa mice. LPL, lipoprotein lipase; VLDL, very-low-density lipoprotein.

To test if impaired lipolysis of triglyceride-rich lipoproteins (TRLs, VLDL, chylomicrons, and their remnants) also might contribute to the postprandial dyslipidemia, we measured the mobilization and activity of lipoprotein lipase (LPL) after intravenous injection of heparin, which releases tissue LPL into the plasma. Released LPL enzyme activity in the plasma did not differ in MPS IIIa and wildtype mice (Fig. 3*A*). Mobilization of LPL resulted in rapid reduction of plasma TGs in both MPS IIIa and wildtype mice under fasting conditions (Fig. 3*B*) and after a corn oil gavage (Fig. 3*C*). These results suggest that LPL mobilization and lipolytic capacity is normal in the mutant mice.

To determine if impairment of hepatic clearance of TRLs from the circulation occurred, we challenged mice with human VLDL and measured its disappearance by ELISA using a monoclonal antibody specific to human apolipoprotein B. The injected particles cleared with similar kinetics as in the wildtype mice (Fig. 3*D*; area under the curve = 6800 ± 300 for MPS IIIa mice *versus* 5900 ± 700 for the control, *p* = 0.26).

### Increased lipid absorption may be due to elevated DPP-IV

Increased intestinal lipid absorption can be a consequence of decreased circulating incretins, such as GLP-1, that inhibit hyperphagia and lipid absorption (14). Attempts to measure GLP-1 levels did not meet with success. Previous studies have reported that patients with MPS present with increased plasma DPP-IV levels, the enzyme responsible for incretin degradation (15, 16). Like human patients we observed a significant 1.2-fold increase in DPP-IV activity in plasma obtained from MPS IIIa mice compared with controls (Fig. 3*E*). Overall, the data suggest that the postprandial dyslipidemia in MPS IIIa is not a consequence of changes in lipolysis or aberrant hepatic TRL clearance but more likely a result of decreased incretin activity due to elevated DPP-IV activity with resultant increase in nutrient absorption.

### Enhanced distribution of postprandial lipids to brown adipose tissue

**Figure 4:**
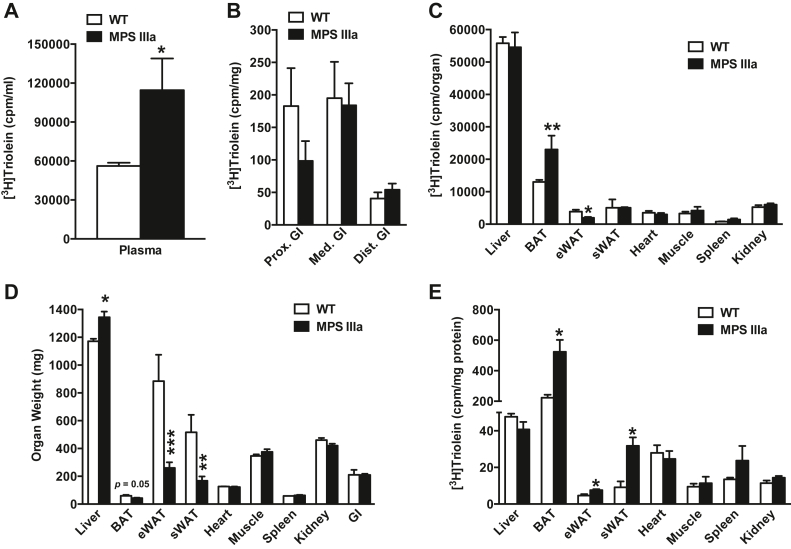
**Increased plasma lipid accumulation and uptake in brown adipose tissue in MPS IIIa mice.** Overnight-fasted mice were given by oral gavage a bolus of [^3^H]retinol mixed with corn oil. After 3 h, blood was obtained by cardiac puncture and plasma was assayed for radioactivity by liquid scintillation counting (*A*). Subsequently, the mice were euthanized and dissected. Individual organs were homogenized and assayed for radioactivity. Counts per organ are reported (*B* and *C*). Organs were weighed (*D*) and counts per milligram of tissue protein was determined (*E*). Each value represents the average ± SEM (n = 3 per group). ∗*p* < 0.05, ∗∗*p* < 0.01, and ∗∗∗*p* < 0.001 compared with wildtype mice.

To examine the fate of dietary fat, mice were orally gavaged with [^3^H]triolein and corn oil. Plasma [^3^H]triolein levels were elevated in the mutant compared with wildtype mice (Fig. 4*A*), consistent with postprandial studies (Fig. 1*D*). Simultaneous reduction in the accumulation of tissue [^3^H]triolein was observed in the proximal small intestine, the primary site of lipid absorption (Fig. 4*B*). [^3^H]triolein uptake in most tissues did not differ in the mutant compared with the wildtype, with the exception of adipose tissue. Uptake in BAT was 2.4-fold greater compared with wildtype mice (Fig. 4*C*), whereas lipid uptake into epididymal white adipose tissue (eWAT) was significantly less in the mutant. Organ mass of eWAT and subcutaneous WAT (sWAT) was lower in the mutant (Fig. 4*D*), resulting in significantly enhanced uptake of [^3^H]triolein per mg tissue protein (Fig. 4*E*). No significant difference in body weight was observed between wildtype and MPS IIIa mice (26.8 ± 1.1 g *versus* 25.1 ± 1.1 g, respectively; *p* = 0.12) during necropsy and after removal of urine from the hyperextended bladder in MPS IIIa mice (13, 17). These findings show that adipose tissue in MPS IIIa mice is metabolically more active compared with wildtype mice.

### MPS IIIa mice depend on lipids for thermogenesis and energy expenditure

**Figure 5:**
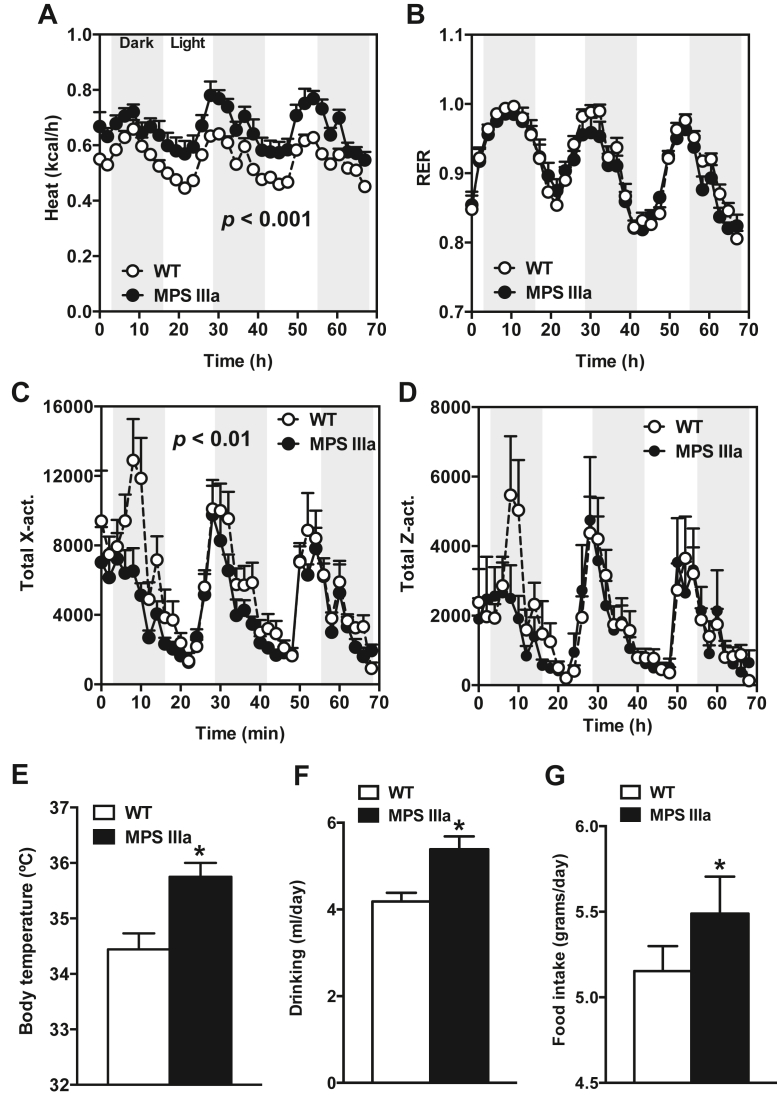
**Increased Thermogenesis and Energy Expenditure in MPS IIIa mice.***A* and *B*, average energy expenditure (heat; A) and respiratory exchange ratio (RER; B) (n = 6 per group). *C*–*D*, activity levels in *dark* and *light cycles* (n = 3–4 per group). *E*, body temperature in overnight-fasted 4-month-old wildtype and MPS IIIa mice was measured using a rectal probe (n = 8–12 per group). *F*–*G*, drinking (*F*) and food intake (*G*) of 4-month-old wildtype and MPS IIIa mice on a chow diet. *E*–*G*, Each value represents the average ± SEM. ∗*p* < 0.05 compared with wildtype mice.

Brown adipose tissue is responsible for nonshivering thermogenesis. High-caloric nutrients, such as lipids and glucose, are metabolized, and the uncoupling of electron transport from ATP synthesis results in heat generation. Indirect calorimetry measurements showed that MPS IIIa mice have significantly increased energy expenditure (expressed as heat) compared with wildtype controls (Fig 5*A*). The respiratory exchange ratio, which measures CO_2_ production and O_2_ consumption, did not significantly differ, indicating no overall shift in the nutrient source used for energy production (Fig. 5*B*). The increased heat production also was not explained by differences in physical activity, as measured by recording movement (Fig. 5, *C* and *D*). Correspondingly, MPS IIIa mice presented with elevated core body temperatures compared with control mice (Fig. 5*E*). Food and water consumption were both significantly increased as well (Fig. 5, *F* and *G*). A standard blood panel revealed increased blood urea nitrogen and chloride levels, while alanine aminotransferase, aspartate aminotransferase, albumin, creatinine, and bilirubin were reduced (Table S1). The increased food consumption is consistent with an increased energy demand. Changes in drinking behavior are more complex and may simply correlate with increased food intake.

**Figure 6:**
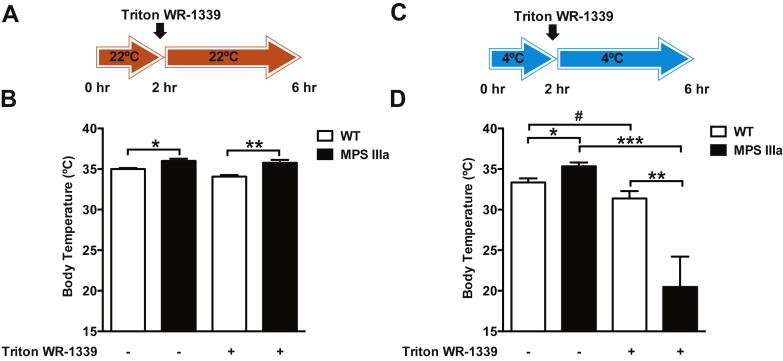
**MPS IIIa Mice Require Lipid Fueling to Maintain Core Body Temperature.***A* and *B*, body temperature was measured in overnight-fasted 4-month-old wildtype and MPS IIIa mice 4 h after treatment with or without Triton WR-1339 to inhibit lipolysis (n = 5–8 per group) at 22 °C. *C* and *D*, body temperature was measured in overnight-fasted 4-month-old wildtype and MPS IIIa mice after being housed for 6 h at 4 °C, which included a 4-h Triton WR-1339 treatment to inhibit lipolysis (n = 3–4 per group). Each value represents the average ± SEM. ∗*p* < 0.05, ∗∗*p* < 0.01, and ∗∗∗*p* < 0.001 compared with wildtype mice, # *p* < 0.05 compared with no Triton WR-1339-treated MPS IIIa mice.

To determine the significance of enhanced lipid uptake in BAT and thermogenesis under activating conditions, mice were injected with Triton WR-1339 to block lipolysis and consequently lipid delivery to BAT. Triton WR-1339 treatment had no effect on body temperature when the animals were housed at room temperature but remained elevated in MPS IIIa mice compared with wildtype mice (Fig. 6*A*). However, when the animals were shifted to a cold environment for 6 h (acclimation for 2 h at 4 °C, followed by a 4-h Triton WR-1339 treatment at 4 °C), mutant mice became severely hypothermic and moribund (Fig. 6*B*). Owing to the severe weight loss in MPS IIIa mice induced by this regimen, one mouse died after 6 h, after which the experiment was stopped. No weight loss was observed in wildtype mice. We also measured glycogen levels in liver, skeletal muscle, and heart muscle under fasting conditions to determine how well MPS IIIa mice can access stored glycogen (18). Under fasting conditions MPS IIIa mice presented with significantly higher glycogen content in both liver (2-fold) and skeletal muscle (2.5-fold), whereas cardiac tissue glycogen content remained unchanged (Fig. S1*B*). Glucose tolerance was normal (Fig. S1*A*). These findings indicate that MPS IIIa mice do not properly access their energy stores (*i.e.*, lipids, glucose, and glycogen) and instead rely on exogenous nutrient sources, especially dietary lipids to maintain normothermia and energy homeostasis.

### Impaired autophagy in MPS IIIa BAT is associated with increased mitochondrial content

**Figure 7:**
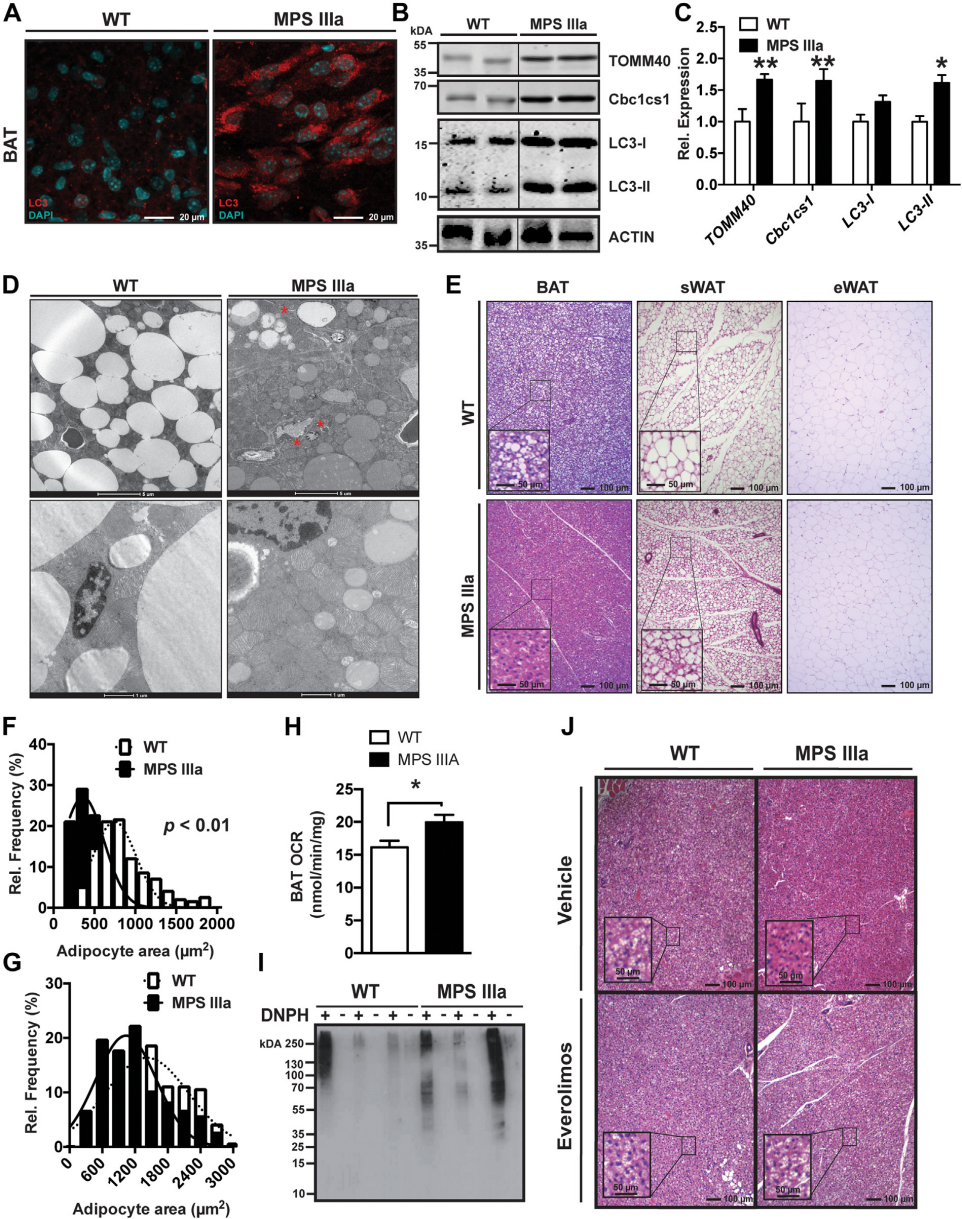
**Impaired Autophagy in MPS IIIa is Associated with Increased Mitochondrial Content in BAT.***A*, representative microphotographs from BAT stained for LC3 (*red*) and nuclei (DAPI; *cyan*) from 6-month-old wildtype and MPS IIIa mice. *B*, representative immunoblots of BAT from 4-month-old wildtype and MPS IIIa mice for expression of mitochondrial markers TOMM40 and cytochrome b-c1 complex subunit 1 (Cbc1cs1) and the autophagy marker LC3. *C*, quantification of BAT immunoblots from 4-month-old wildtype and MPS IIIa for expression of mitochondrial markers TOMM40 and Cbc1cs1 and the autophagy marker LC3 (n = 4/group). *D*, representative electron microscopy images from BAT isolated from 6-month-old MPS IIIa and wildtype controls. *E*, representative microphotographs from H&E-stained BAT, sWAT, and eWAT of 4-month-old wildtype and MPS IIIa mice. *F* and *G*, adipocyte diameter distribution in sWAT (*F*) and eWAT (*G*) (n = 4/group). *H*, oxygen consumption rate securements in BAT slices isolated from 6-month-old wildtype and MPS IIIa mice (n = 3–4/group). *I*, analysis of protein oxidation in lysates from BAT isolated from 6-month-old wildtype and MPS IIIa mice after 2,4-dinitrophenylhydrazine (DNPH) treatment. *J*, representative microphotographs from H&E-stained BAT of 4-month-old wildtype and MPS IIIa mice treated with everolimus (10 mg/kg) or vehicle prior to sacrifice for 5 weeks. Each value represents the average ± SEM. ∗*p* < 0.05 and ∗∗*p* < 0.01 compared with wildtype mice. BAT, brown adipose tissue; eWAT, epididymal white adipose tissue; sWAT, subcutaneous white adipose tissue.

Settembre and coworkers have shown that autophagy flux in MPS IIIa mice is reduced (19, 20). Immunohistochemical analysis of BAT in MPS IIIa mice showed a significant accumulation of LC3-positive autophagosomes (Fig. 7*A*). Western blotting showed that LC3-I and LC3-II levels increased 1.3- and 1.6-fold, respectively, in BAT from mutant mice compared with BAT from wildtype mice (Fig. 7, *B* and *C*). The increased thermogenesis in MPS IIIa mice could be explained by defective autophagy-mediated mitochondrial clearance (mitophagy), which has been implicated in reducing thermogenic adipose tissue activity after cold exposure (21). Indeed, impaired autophagy was associated with a significantly increased accumulation of mitochondria as shown by electron microscopy (Fig. 7*D*). This correlated with a significant accumulation of the mitochondrial markers TOMM40 and cytochrome b-c1 complex subunit 1, (Cbc1cs1; encoded by UQCRC1), which increased 1.7- and 1.6-fold, respectively, in MPS IIIa mice compared with WT controls (Fig. 7, *B* and *C*). We also observed a significant increase in the number of mitophagosomes in MPS IIIa BAT (Fig. S1).

Multilocular beige adipocytes accumulated in sWAT from MPS IIIa mice, indicative of mitochondrial accumulation (Fig. 7*E*, inset). Lipid droplet size and number in BAT (Fig. 7*E*, inset) and sWAT (Fig. 7*F*) mice were significantly reduced, whereas adipocyte size in eWAT showed a trend toward smaller size (Fig. 7*G*). It was not possible to evaluate quantitatively lipid droplet size in BAT because of their high density in tissue from wildtype and mutant mice. The reduction in droplet size and number in white adipose tissue reflects increased mobilization of fat stores, presumably due to higher energy demand.

We next evaluated if the higher density of mitochondria in BAT from MPS IIIa mice correlated with a functional increased in tissue energy metabolism. Using a Clark electrode, we observed a significant 1.2-fold increase in oxygen consumption in whole *ex vivo* BAT slices from MPS IIIa mice compared with BAT from wildtype controls (Fig. 7*H*). The increased mitochondrial content also correlated with elevated oxidative stress in BAT from MPS IIIa mice as measured by increased protein oxidation (Fig. 7*I*).

To determine if impaired mitophagy in MPS IIIa was driving the mitochondrial accumulation we treated MPS IIIa and WT mice with everolimus for 5 weeks, a well-established mTOR inhibitor and activator of autophagy. Treatment of MPS IIIa mice with everolimus was associated with a significant increase in BAT lipid droplet size and number compared with BAT from vehicle-treated MPS IIIa mice (Fig. 7*J*, inset). In fact, BAT from everolimus-treated MPS III a mice had a similar morphology as BAT from vehicle- or everolimus-treated wildtype mice (Fig. 7*J*). Overall, the data suggest that impaired autophagy in BAT from MPS IIIa mice correlates with increased mitochondrial content and BAT activity and energy demand.

### Enzyme replacement therapy reverses lipid, autophagosome, and mitochondria accumulation

**Figure 8:**
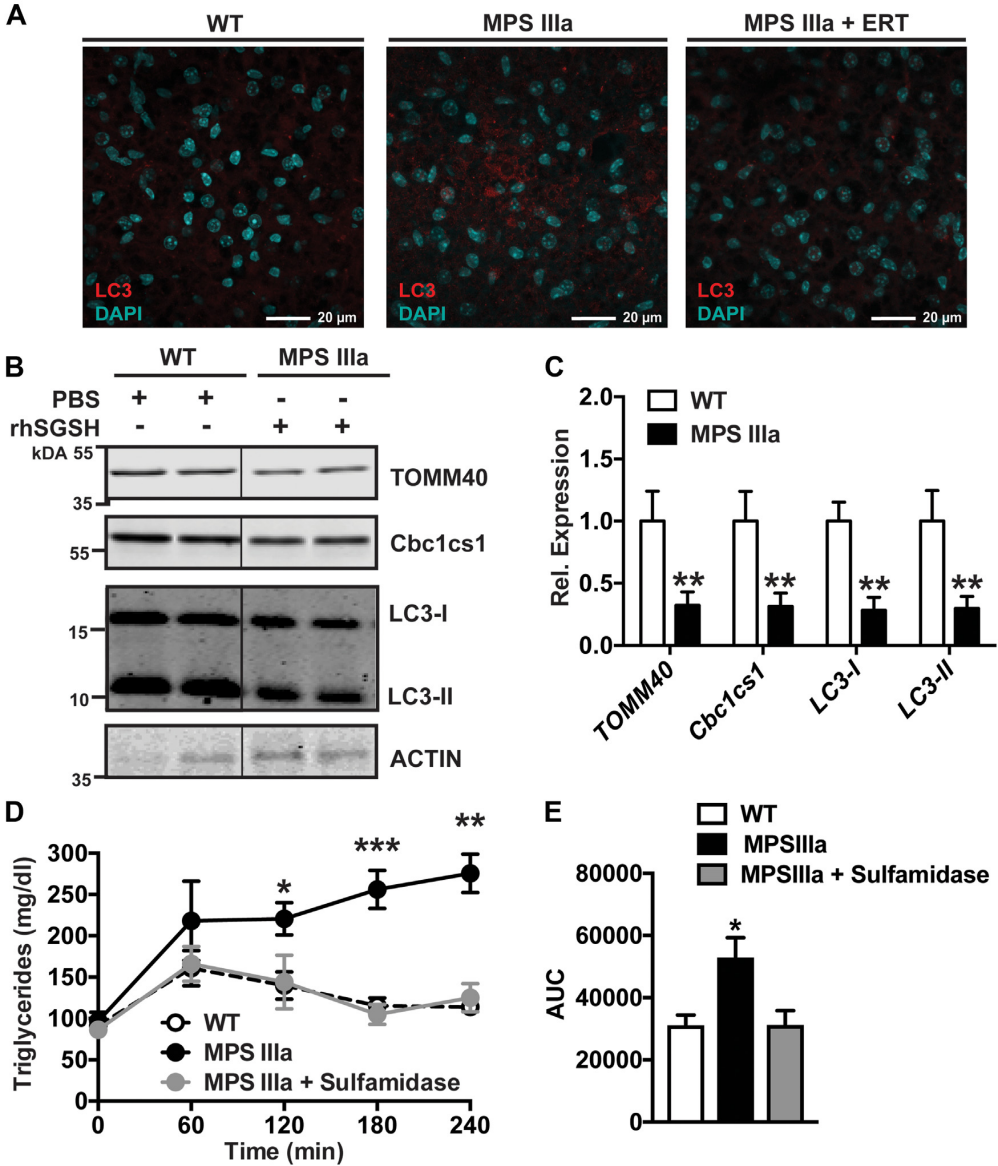
**ERT reverses the hypotriglyceridemia and mitochondrial accumulation of BAT.***A*, representative microphotographs from BAT of 4-month-old wildtype, PBS-treated MPS IIIa, and ERT-treated MPS IIIa mice (recombinant sulfamidase at 1 mg/kg/week for 5 weeks) stained for LC3 (*red*) and nuclei (DAPI; *cyan*). *B*, immunoblot analysis of BAT from wildtype mice and MPS IIIa mice treated with i.v. injections of recombinant sulfamidase (1 mg/kg/week for 5 weeks) for expression of mitochondrial markers TOMM40 and cytochrome b-c1 complex subunit 1 (Cbc1cs1) and the autophagy marker LC3-I and LC3-II. *C*, quantification of BAT immunoblots from 4-month-old wildtype and MPS IIIa for expression of mitochondrial markers TOMM40 and Cbc1cs1 and the autophagy marker LC3 (n = 2–3/group). *D* and *E*, postprandial triglyceride clearance in overnight-fasted wildtype mice, PBS-treated MPS IIIa mice, and ERT-treated MPS IIIa mice (1 mg/kg/week for 5 weeks, i.v.) after giving 200 μl of corn oil by oral gavage. Each value represents the average ± SEM. ∗*p* < 0.05, ∗∗*p* < 0.01, and ∗∗∗*p* < 0.001 compared with wildtype mice. BAT, brown adipose tissue; ERT, enzyme replacement therapy.

We also investigated if the hyperlipidemia and BAT phenotype could be reversed by enzyme replacement therapy (ERT). Adult MPS IIIa mice (4 months) received ERT *via* intravenous administration of recombinant human sulfamidase (1 mg/kg/week for 5 weeks). As a control, wildtype mice were treated with an equal amount of PBS. Analysis of BAT after ERT revealed a dramatic reduction in the expression of LC3-I and LC3-II (5-fold) and mitochondrial markers TOMM40 (3-fold) and cytochrome b-c1 complex subunit 1 (Cbc1cs1; 3.3-fold) compared with PBS-treated wildtype mice (Fig. 8, *A*–*C*). The LC3-I and LC3-II levels were not different in BAT from MPS IIIa mice compared with BAT from wildtype mice after ERT as measured by the LC3-II/LC3-I ratio (vs. 0.55 ± 0.05 vs. 0.61 ± 0.03, respectively, *p > 0.05*). ERT in MPS IIIA mice fully restored postprandial lipid clearance to wildtype levels compared with the observed postprandial dyslipidemia in untreated MPS IIIa mice (Fig. 8, *D* and *E*). The data indicate that dyslipidemia and BAT hyperactivity can be reversed by ERT in the periphery.

## Discussion

**Figure 9:**
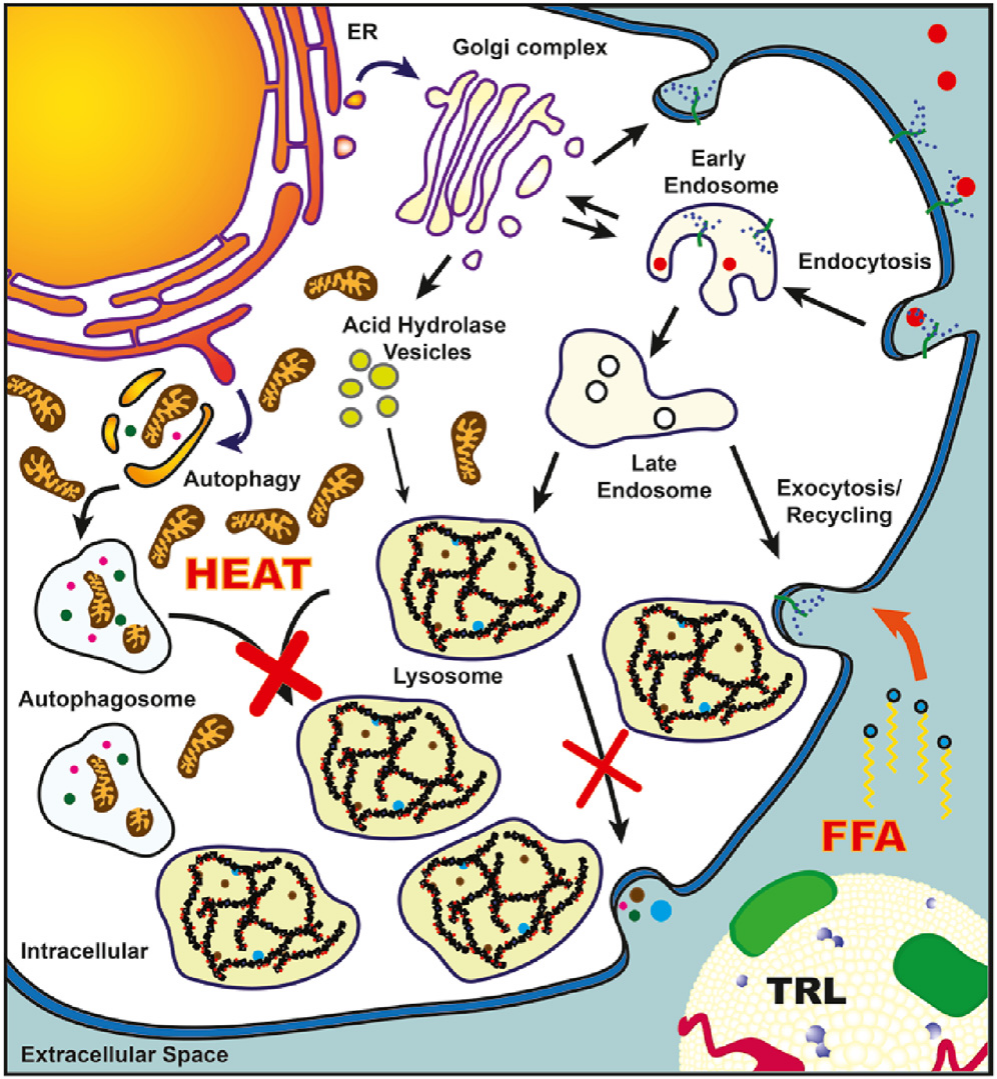
**Model for Increased Energy Expenditure in Lysosomal Storage Disease.** lysosomal storage disease, such as MPS IIIa, results in various developmental and physiological complications, including cachexia. Loss of lysosomal function in MPS IIIa mice increases mitochondria accumulation due to impaired autophagosome–lysosome fusion. As a result, this increases brown adipose tissue activity and overall energy expenditure and is associated with a dramatic shift in energy reliance on exogenous lipids, with ensuing hypertriglyceridemia. The findings provide further evidence that the lysosome is critical for maintaining mitochondria content in brown adipose tissue and consequently energy expenditure in patients with MPS IIIa and other lysosomal storage disease. FFA, free fatty acid; triglyceride-rich lipoprotein.

Cachexia is often a terminal complication in lysosomal storage disorders (LSD) and is associated with more severe and advanced disease stages (22, 23, 24, 25). To study potential causes of the negative energy balance associated with cachexia, we assessed the impact of sulfamidase deficiency and heparan sulfate storage on lipid utilization in MPS IIIa mice. We identified that impaired autophagy in LSD is associated with hyperactivity in BAT and consequential hyperthermia. This process increases energy expenditure and is associated with a dramatic shift in energy reliance on exogenous lipids, with ensuing hypertriglyceridemia (Fig. 9).

Alterations in fasting TG levels have been reported in multiple murine MPS models, including MPS I and MPS IIIb, but not for MPS IIIa (6). However, the more relevant postprandial TG response was not previously investigated for any MPS animal models. We observed postprandial hypertriglyceridemia in MPS I, IIIa, and IIIb mice, most dramatically in the two Sanfilippo syndromes. The variation in response in the different MPS disorders suggests that storage of different classes of GAGs leads to different metabolic consequences. The Sanfilippo syndromes affect processing of the nonreducing end hexosamine residue in heparan sulfate, resulting in selective storage of heparan sulfate. The other MPS disorders (MPS I, II, and VII) result in storage of both chondroitin/dermatan sulfate as well as heparan sulfate. The impact of storing different subtypes of GAGs on lipid homeostasis and adipose tissue is unknown but may reflect differences in the degree of GAG accumulation in WAT, BAT, and intestinal enterocytes.

Woloszynek *et al.* reported mild fasting hypotriglyceridemia for MPS I and MPS IIIb, contrasting the findings reported here (6). This discrepancy might be explained by different housing regimes (food and temperature). The postprandial dyslipidemia findings reported here are in accordance with a recent metabolic study of serum samples from human MPS III and MPS IIIb subjects, in which 70% of the elevated metabolites were found to be TGs and their derivatives, such as long-chain fatty acids, polyunsaturated fatty acids, and monoacylglycerols (26).

Elevated plasma TGs (hypertriglyceridemia) results from an imbalance between *de novo* TG synthesis in the liver (VLDL), intestinal absorption of dietary fats (chylomicrons), lipolysis in the peripheral circulation (mediated mostly by LPL), and hepatic clearance (27). No differences were seen in LPL mobilization and activity, hepatic TG-remnant clearance, or VLDL production in MPS IIIa versus control mice. However, an increase in intestinal lipid absorption and secretion occurred. It is well established that even under our moderate fasting conditions (5 h) that increases intestinal lipid absorption will present as a persistent increase in plasma triglyceride levels. Longer fasting regimens (16 h) of MPS IIIA mice resulted in reduced plasma triglyceride levels (0 h time point in Fig. 1, *D* and *G*), not different from wildtype mice. This observation is consistent with the concept that the hypertriglyceridemia was a consequence of increased intestinal lipid absorption and not altered lipoprotein distribution and metabolism under fasting conditions.

MPS IIIA mice present with slightly elevated blood urea nitrogen levels and moderately lower creatinine and alanine aminotransferase levels. This is suggestive for increased kidney stress in MPS IIIA. However, these levels are not consistent with kidney failure, nor has kidney failure been reported in MPS IIIA mice before. The increased plasma triglyceride levels were not due to increased liver VLDL production or impaired liver or systemic clearance of lipoproteins. Thus, the increased plasma triglyceride levels in MPS IIIA are consistent with increased intestinal lipid absorption and do not seem to be a consequence of liver or kidney failure. However, this does not exclude the possibility that the increased stress on the kidney and liver induced by lysosome storage can result in some axis whereby the liver and/or kidney communicate with the gut to absorb more lipids.

In the gut, dietary lipids are hydrolyzed into free fatty acids (FFAs) and free cholesterol in the intestinal lumen and taken up by enterocytes in the proximal small intestine (28). Enteric FFA absorption occurs passively when luminal FFA concentrations are high, as in the case of a corn oil gavage, or actively at lower luminal FFA concentrations by receptors such as CD36 and other FFA transport proteins. In contrast, cholesterol absorption is exclusively an active process requiring Niemann-Pick C1-like 1 (NPC1L1) receptor and ABCG5/ABCG8 dimers on enterocytes (28, 29). No significant change in intestinal permeability was noted, and lipids did not accumulate in the tissues, suggesting that enterocytes in the MPS IIIa mice assemble or secrete chylomicron particles more efficiently (29). This gain-of-function phenotype is rather counterintuitive in the context of this progressive disabling disease. The mechanism underlying enhanced lipid secretion from the intestine is unknown but may be related to apoB production, chylomicron assembly, or a decrease in adipocyte-mediated production of ANGPTL4, a known inhibitor of LPL activity and intestinal lipid absorption.

The hypertriglyceridemia in MPS mice was associated with increased BAT lipid uptake and hyperthermia, resulting in increased energy expenditure. Previous studies reported that several murine models of MPS present with increased energy expenditure due to inefficient recycling of sugar precursors generated by the degradation of glycans in the lysosome (6, 7). Our observations diverge from this hypothesis as we observed that MPS IIIa mice have increased energy expenditure predominantly due to hyperactive BAT and increased beige adipocyte content in sWAT (10, 30). Second, normal glucose tolerance tests suggest that increasing glucose influx would not overcome the energy deficits imposed by BAT and beige adipose tissue hyperactivity. Instead, we provide evidence suggesting that the inability of MPS IIIa mice to access energy pools (lipids, glucose, and glycogen) drives the increased demand for exogenous lipids to overcome the energy deficit. This becomes apparent when MPS IIIa mice are exposed to cold stress, as they fail to maintain normal body temperature when lipid distribution to BAT is hindered. Under these extreme conditions normal mice degrade stored lipids and glycogen to fuel oxidative tissues, including BAT (18, 31, 32). However, MPS IIIa mice maintain glycogen accumulation in liver and skeletal muscle, at least under starvation conditions, confirming that glycogen is an inaccessible secondary storage metabolite in MPS IIIa, which was suggested previously (33). The deficit in autophagy manifested by MPS IIIa mice and other models of MPS may reflect defective glycophagy as well as mitophagy as reported previously (19, 20).

A recent study presented compelling data underscoring the relevance of autophagy in thermogenic adipose tissue. Altshuler-Keylin *et al* showed that autophagy-mediated mitochondrial clearance controls beige adipocyte maintenance (21). Genetic inactivation of mitophagy prevented beige adipocyte loss after withdrawal of external stimuli, maintaining a high thermogenic capacity and protecting against diet-induced obesity and insulin resistance (21). The importance of mitophagy in BAT control was unclear, but the authors alluded to its possible relevance in older mice. This led us to evaluate autophagy in BAT from starved MPS IIIa mice, and a dramatic accumulation of LC3-positive autophagosomes and mitochondria was observed. The relevance of deficient autophagy for the mitochondrial accumulation in MPS IIIa BAT is further supported by the fact that treatment with the mTOR inhibitor, everolimus, reverted the BAT phenotype in MPS IIIa mice. These observations are in accordance with the reported accumulation of mitochondria in BAT during whitening (at thermoneutrality) in mice with BAT-specific inactivation of Transcription Factor EB (34), the master transcriptional regulator of lysosomal and autophagosomal biogenesis (35). The increased mitochondria content in Transcription Factor EB–deficient BAT after reversing thermogenic activation was independent of mitochondria biogenesis but correlated with increased mitochondrial trapping in autophagosomes (34). Our data provide further evidence that indeed impaired autophagy and lysosomal activity in BAT associates with accumulation of mitochondria and the consequent hyperthermia and loss of adipose tissue mass. Impaired autophagosomal–lysosomal fusion has been reported previously in hepatocytes and neurons isolated from MPS IIIa mice (19, 20). We also observed an increase in mitophagosomes in BAT from MPS IIIa mice (Fig. S1).

Systemic administration of ERT normalized postprandial lipid clearance, BAT hyperactivity, and autophagosome accumulation in the studies reported here. The fact that systemic ERT does not correct lysosomal storage accumulation in the brain or dysfunctional mitochondria in MPS IIIa neurons (19) suggests that the defect in lipid homeostasis is not caused by central nervous system manifestations of the disease, which progressively appear from birth (36).

Based on the data reported here, impaired autophagy in BAT results in progressive accumulation of mitochondria with consequent hyperthermia and loss of adipose tissue mass to fuel BAT (Fig. 9). Such an energy imbalance will result in dramatic loss of adipose tissue if inadequate dietary fat is ingested. A chronic negative energy balance can contribute to a state of cachexia, a condition that is commonly associated with MPS disorders despite adequate basal energy intake (22, 23). Cachexia, or wasting, is more common in patients with severe forms of MPS or in patients who remained undiagnosed for a prolonged period of time (22, 23, 37). BAT hyperactivity and WAT “browning” are known to contribute to cancer-induced cachexia and weight loss in children with malignant cancers (38, 39, 40). Although it is conceivable that BAT hyperactivity will increase the energy demand in MPS IIIa mice, it is very likely that mitochondrial accumulation in other oxidative tissues will also contribute to the progressive energy drain in LSD. Furthermore, it is important to consider that the energy expenditure increase due to BAT activity in MPS IIIa might eventually disappear in time if organelle biogenesis is also affected.

Future experiments need to determine to what extent lysosomal storage of heparan sulfate in BAT and the associated hyperactivity of BAT in MPS IIIA mice contributes to the wasting phenotype. Crossing MPS IIIa mice in an UCP1-deficient background or breeding and maintaining them in a thermoneutral environment could provide a basis for neutralizing BAT function on long-term survival and energy homeostasis in MPS disorders. However, the causative role of hyperactive BAT for cachexia in MPS IIIA is difficult to assess even with the suggested thermoneutral experiment. We do not expect increased BAT activity to be the sole driver of the MPS IIIA-associated cachexia. Other factors, including, but not restricted to, kidney damage, liver stress, and neurodegeneration, are most likely also important contributing factors to cachexia in MPS IIIA. Identifying their relative contribution to cachexia development in MPS IIIA is a very interesting question. However, MPS IIIA disease manifestation is complex, progressive, and multifactorial as mice (and patients) have multiple organ deficiencies that arise from a different degree of lysosomal storage in different cell types at different times during development and aging. Hence, identification of BAT or other affected tissues as the causative role of MPS IIIA cachexia is a very complex question beyond the scope of the current study. Future extensive analyses using organ-specific enzyme replacement modalities in combination with environmental thermoneutrality need to be designed to identify the relative contributions of pathophysiological manifestations in the brain, kidney, liver, BAT, and others organ for the onset of cachexia in MPS IIIA.

In conclusion, we hypothesize that increased BAT activity and persistent increase in energy demand in MPS IIIa mice can contribute to the progressively elevated energy demand observed in MPS IIIa and other LSD patients. Our findings also warrant additional studies in humans to determine their clinical relevance and to better understand the etiology of the increased intestinal lipoprotein secretion in the gut and its connection to BAT hyperactivity.

## Experimental procedures

### Mice and animal husbandry

Mice bearing a hypomorphic allele in sulfamidase (*Sgsh*, B6.Cg-Sgshmps3a/PstJ) (13) and a null allele in alpha-N-acetylglucosaminidase (*Naglu*) (B6.129S6-Naglutm1Efn/J) were purchased from Jackson Laboratory. All animals were housed and bred in vivaria approved by the Association for Assessment and Accreditation of Laboratory Animal Care located in the School of Medicine, UCSD, following standards and procedures approved by the UCSD Institutional Animal Care and Use Committee. In all cases heterozygous female mice were used for breeding and offspring, which were weaned at 3 weeks of age, maintained on a 12-h light cycle, and fed *ad libitum* with water and standard rodent chow (Harlan Teklad). Mice were treated *via* oral gavage with 100 μl vehicle (12.5% Cremophor EL and 12.5% ethanol in water) or everolimus (10 mg/kg body weight in 100 μl; Fisher Scientific) 3 times per week for 5 weeks as described (41). Enzyme replacement was performed on 8-week-old mice by i.v. treatment for 5 weeks with 1 mg/kg/week of human recombinant sulfamidase or 200 μl PBS as described (42).

### Plasma lipids and lipoprotein distribution

Blood was obtained by cardiac puncture from mice fasted for 16 h, or *via* the tail vein using microvette CB 300 capillaries (Sarstedt) from mice fasted for 5 h. Plasma cholesterol and TG levels were measured using commercially available kits (Sekesui). Lipoprotein profiles were performed on pooled plasma from six mice per genotype. Lipoproteins in 200 μl pooled plasma samples were separated by fast performance liquid chromatography (FPLC) gel filtration on a Superose 6 column and cholesterol and TGs were determined in each fraction.

### Hepatic VLDL-TG secretion and intestinal lipid absorption

Mice were fasted for 5 h prior to a tail vein injection of Triton WR-1339 (10% solution in PBS; Sigma-Aldrich) at a dose of 0.5 mg/g body weight. Plasma was collected by tail bleeding at 1, 15, 30, 60, and 120 min after injection. To measure intestinal lipid absorption, fasted mice received an intragastric load of corn oil (10 μl/g body weight). One minute later mice received an intravenous injection of Triton WR-1339 (0.5 mg/g body weight, 10% solution in PBS; Sigma-Aldrich) to block plasma triglyceride hydrolysis (43). Blood samples (60 μl) were drawn by tail bleeding before gavage (time 0) and 1, 2, and 3 h after gavage. Plasma TG was measured as described above.

### Postprandial TG response

Mice were fasted for 16 h prior to receiving an intragastric load of corn oil (10 μl/g body weight). Plasma was collected by tail bleeding for TG measurements at time points 0, 60, 120, 180, and 240 min after injection. Postprandial lipid accumulation in organs was evaluated as described (44). Briefly, mice were fasted for 16 h prior to receiving an intragastric fat load of 200 μl corn oil containing 40 μCi glycerol-tri-[1-^3^H]oleate (Amersham Biosciences) *via* oral gavage. After 2 h, blood was removed by cardiac puncture and the animal was perfused with 10 ml of PBS containing 10 units of heparin before harvesting organs. Organs were solubilized in Solvable (PerkinElmer), and radioactivity was counted by liquid scintillation spectrometry.

### VLDL clearance and lipoprotein lipase activity

Clearance of human VLDL was performed by measuring the level of human apoB-100 present in plasma at timed intervals following intravenous injection of human VLDL purified from fasted, healthy donors. Human apoB-100 was measured by ELISA using mAb MB47, which binds human but not murine apoB-100, exactly as described (45). Intravenous injection of heparin (50 U per mouse) was used to release lipoprotein lipase into the plasma. Blood samples were taken by retro-orbital bleeding prior to and at various times after injection. Enzyme activity in plasma or tissues was determined using sonicated radiolabeled triolein substrate ([9,10(n)-^3^H]triolein, ∼0.5 mCi/ml; PerkinElmer) and calculated as nanomoles of triolein hydrolyzed per minute (*i.e.* mU activity) for each sample.

### Total gut transit test and *in vivo* intestinal permeability

Carmine (200 μl; 3 mg in 0.5% methylcellulose) was orally administered to each mouse. Mice were then returned to individual cages and placed on a white sheet of paper. The time taken for excretion of the first colored feces was recorded in 4 to 5 animals of each genotype. Intestinal barrier permeability was measured in fasted mice after administration of FITC-dextran by gavage (4 kDa; 600 mg/kg body weight; 200 μl; Sigma-Aldrich). Blood was collected 4 h later by tail bleeding. The serum concentration of the FITC-dextran was determined using a fluorimeter (PerkinElmer Life Sciences) with an excitation wavelength of 490 nm and an emission wavelength of 530 nm. Serial-diluted FITC-dextran was used to generate a standard curve.

### Indirect calorimetry

Mice were placed into Comprehensive Lab Animal Monitoring System (CLAMS; Columbus Instruments) metabolic cages to adapt to their surroundings for 48 h. Rates of O_2_ consumption (VO_2_; ml/kg/h) and CO_2_ production (VCO_2_) were measured for each chamber every 17 min throughout the study and used to calculate the respiratory exchange ratio. Energy expenditure was calculated as VO_2_ × (3.815 + [1.232 × (VCO_2_/VO_2_)]). The energy expenditure was not corrected for body weight due the significant bladder urine retention phenotype of MPS IIIa mice (ranging between 1–2 ml) significantly affecting the actual body weight measurements (13, 17). Activity was recorded using infrared beams on axes (X and Z) and presented as the number of beam breaks per axis during an interval.

### Oxygen consumption assay

Direct *ex vivo* tissue respiration in BAT was performed using a Clark electrode system (Hansatech Oxytherm). Freshly isolated tissue was immediately sliced (2 mm) in respiration buffer (1.5 mM pyruvate, 25 mM glucose, and 2% bovine serum albumin) and placed in the Clark electrode chambers. The oxygen consumption rate was normalized to the tissue weight measured at the end of the reading.

### DPP-IV enzyme assay

DPP-IV enzyme assays were performed in 1-ml reaction volumes. A total of 15 μl plasma was added to 485 μl 0.1 M Tris–HCl, pH 8.0, and 500 μl of 1 mM Gly-Pro-p-nitroaniline hydrochloride substrate (Sigma-Aldrich) prepared in 0.1 M Tris–HCl, pH 8.0 was added at timed intervals. Absorbance was measured at 405 nm at 30 and 60 min, and the amount of nitroaniline produced over the 30-min period was calculated from a standard curve using p-nitroaniline (Sigma-Aldrich). Specific activity was expressed as nmol/min/ml of plasma.

### Glycogen determination

Glycogen was prepared from mouse skeletal muscle or liver by boiling the tissue in 30% potassium hydroxide followed by filtration to remove floating fat. The glycogen was precipitated with ethanol and redissolved in water. Glycogen was quantitated by measuring glucose equivalents after digestion with amyloglucosidase.

### Western blotting

Tissues were homogenized in RIPA buffer containing protease inhibitors (Roche). For each sample, 20 μg protein was resolved on a 4% to 12% Bis-Tris NuPage gel (Thermo Fisher) and transferred to polyvinylidene fluoride membrane (Bio-Rad Laboratories). Blots were incubated with antibodies to TOMM40, cytochrome b-c1 complex subunit 1 (Proteintech), LC3 (MBL International), and β-actin (Sigma-Aldrich). Protein oxidation was measured in 35 μg whole BAT lysate using the OxyBlot Protein Oxidation Detection Kit (Millipore Sigma). Bands were visualized on an Odyssey Infrared imaging system (Li-Cor Biosciences) as described (46).

### Immunohistochemistry

Mice were anesthetized using a mixture of ketamine/xylazine and transcardially perfused Dulbecco’s PBS followed by 4% phosphate-buffered paraformaldehyde. Tissues were postfixed overnight at 4 °C and embedded in paraffin. Sections (15 μm) were mounted directly onto glass slides and stained with an anti-LC3 antibody (MBL International) as described (36). Species-specific Alexa Fluor 647–conjugated secondary antibodies were used to visualize primary antibody staining and eliminate autofluorescence of the tissues. Confocal images were collected on a Nikon Ti microscope equipped with the Nikon A1R confocal system using a high NA 100X oil objective. The ND acquisition tool was used to acquire Z stacks in three channels.

### Electron microscopy

Interscapular BAT and small tissue fragments were immersed in modified Karnovsky’s fixative (2.5% glutaraldehyde and 2% paraformaldehyde in 0.15 M sodium cacodylate buffer, pH 7.4) for at least 4 h, postfixed in 1% osmium tetroxide in 0.15 M cacodylate buffer for 1 h, and stained *en bloc* with 2% uranyl acetate for 1 h. Samples were dehydrated in ethanol, embedded in Durcupan epoxy resin (Sigma-Aldrich), sectioned at 50 to 60 nm on a Leica UCT ultramicrotome, and picked up on Formvar and carbon-coated copper grids. Sections were stained with 2% uranyl acetate for 5 min and Sato's lead stain for 1 min. Grids were viewed using a JEOL 1200EX II (JEOL) transmission electron microscope and photographed using a Gatan digital camera (Gatan) or viewed using a Tecnai G2 Spirit BioTWIN transmission electron microscope equipped with an Eagle 4k HS digital camera (FEI). Mitophagosomes were quantified as described by Duan *et al*. (47).

### Statistical analysis

All data were analyzed by Student’s *t* test or two-way ANOVA and presented as mean ± SEM. Statistical analyses were performed using Prism software (version five; GraphPad Software). *p* Values less than 0.05 were considered significant.

## Data availability

All data are available in the main article or the supporting information.

## Supporting information

This article contains supporting information.

## Conflict of interest

The University of California, San Diego and J. D. E. have a financial interest in TEGA Therapeutics, Inc. The terms of this arrangement have been reviewed and approved by the University of California, San Diego in accordance with its conflict-of-interest policies. B. E. T and C. A. G. are employees of TEGA Therapeutics, Inc.

## Supporting information

Gordts MPS Paper Supporting Information JBC- third revision

Gordts MPS MAin Table I JBC - second revision
