# Supplementary material for: Impaired mitophagy in Sanfilippo a mice causes hypertriglyceridemia and brown adipose tissue activation
Source: J Biol Chem. 2022 Jun 22;298(8):102159. doi: 10.1016/j.jbc.2022.102159 (PMC9364035; doi:10.1016/j.jbc.2022.102159)
Supplement: Gordts MPS Paper Supporting Information JBC- third revision [file mmc1.docx]

**Supporting Information**


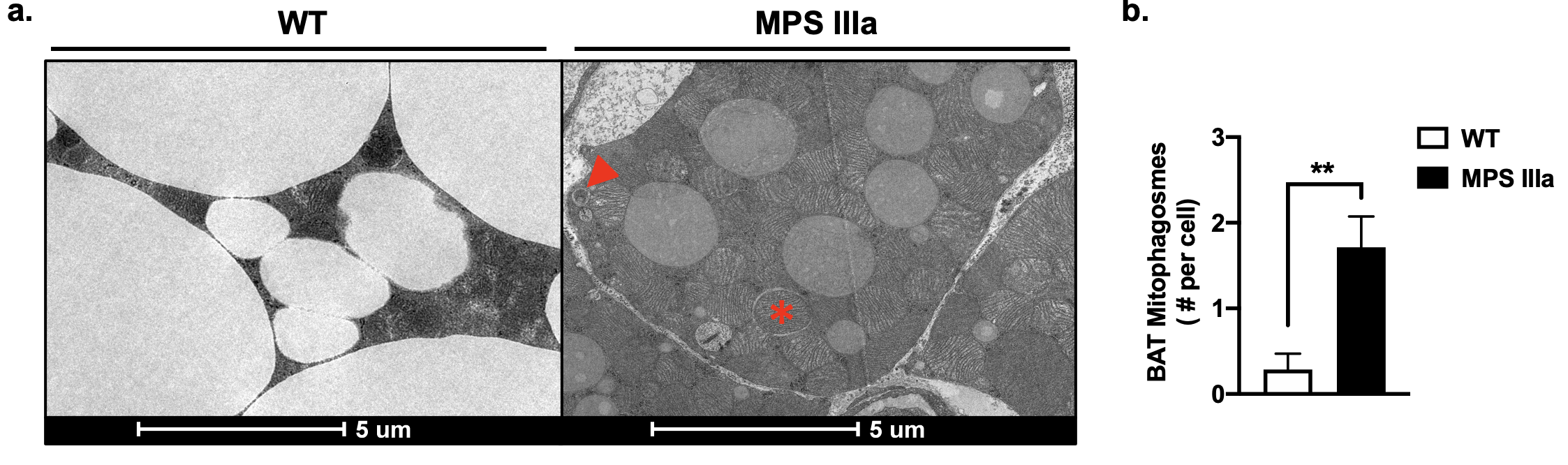


**Supporting Figure 1. Mitophagosome quantification in wildtype and MPS IIIa BAT.** a, Representative EM microphotographs of BAT from wildtype (WT) and MPS IIIa hypomorph mice. The red star and red arrow indicate mitophagosomes. b, Quantification of the number of mitophagosomes per brown adipocytes (n = 7 cells per group) obtained from 6-month-old wildtype (n = 3) and MPS IIIa (n = 2) mice. Each value represents the average ± SEM. ** *p* < 0.01 compared to wild type mice.
