# Supplementary material for: Impaired mitophagy in Sanfilippo a mice causes hypertriglyceridemia and brown adipose tissue activation
Source: J Biol Chem. 2022 Jun 22;298(8):102159. doi: 10.1016/j.jbc.2022.102159 (PMC9364035; doi:10.1016/j.jbc.2022.102159)
Supplement: Gordts MPS MAin Table I JBC - second revision [file mmc2.docx]

**Supporting Information**

**Supporting Table I.** **Plasma Biochemistry Panel**

Each value represents the average ± SEM (n = 4 per group). ALT, Alanine Aminotransferase; AST, aspartate aminotransferase; BUN, blood urea nitrogen.

|  |  | **Wild type** | | |  | **MPS IIIa** | | |
| --- | --- | --- | --- | --- | --- | --- | --- | --- |
|  |  |  |  |  |  |  |  |  |
| **Albumin** | *g/dL* | 4.3 | ± | 0.1 |  | 3.8 | ± | 0.1^a^ |
| **Alkaline Phosphatase** | *U/L* | 110.0 | ± | 14.6 |  | 122.7 | ± | 11.4 |
| **ALT** | *U/L* | 30.0 | ± | 2.0 |  | 22.7 | ± | 0.5^a^ |
| **Anion Gap** |  | 39.0 | ± | 0.8 |  | 35.0 | ± | 2.0 |
| **AST** | *U/L* | 160.0 | ± | 30.0 |  | 116.7 | ± | 9.7 |
| **Bicarbonate** | *mmol/L* | 14.0 | ± | 0.4 |  | 14.5 | ± | 0.9 |
| **Bilirubin Total** | *mg/dL* | 0.13 | ± | 0.00 |  | 0.11 | ± | 0.01^a^ |
| **BUN** | *mg/dL* | 23.0 | ± | 1.6 |  | 32.2 | ± | 2.1^a^ |
| **Calcium** | *mg/dL* | 10.2 | ± | 0.1 |  | 10.1 | ± | 0.1 |
| **Chloride** | *mmol/L* | 99.3 | ± | 0.4 |  | 103.8 | ± | 1.0^a^ |
| **Creatinine** | *mg/dL* | 0.14 | ± | 0.00 |  | 0.12 | ± | 0.01^a^ |
| **Glucose** | *mg/dL* | 60.0 | ± | 8.0 |  | 76.1 | ± | 17.7 |
| **Potassium** | *mmol/L* | 7.8 | ± | 0.3 |  | 8.7 | ± | 0.4 |
| **Sodium** | *mmol/L* | 152.3 | ± | 0.6 |  | 153.3 | ± | 0.5 |
| **Total Protein** | *g/dL* | 5.8 | ± | 0.0 |  | 5.6 | ± | 0.1 |
|  |  |  |  |  |  |  |  |  |

^a^ Values indicate a significant difference (*p* < 0.05) from wild type animals.
